# Effects of Quinoa Flour on Wheat Dough Quality, Baking Quality, and *in vitro* Starch Digestibility of the Crispy Biscuits

**DOI:** 10.3389/fnut.2022.846808

**Published:** 2022-04-13

**Authors:** Yanrong Ma, Daying Wu, Lei Guo, Youhua Yao, Xiaohua Yao, Zhonghua Wang, Kunlun Wu, Xinyou Cao, Xin Gao

**Affiliations:** ^1^State Key Laboratory of Crop Stress Biology in Arid Areas and College of Agronomy, Northwest A&F University, Yangling, China; ^2^State Key Laboratory of Plateau Ecology and Agronomy, Qinghai Key Laboratory of Hulless Barley Genetics and Breeding, Qinghai Subcenter of National Hulless Barley Improvement, Qinghai University, Xining, China; ^3^National Engineering Laboratory for Wheat and Maize, Key Laboratory of Wheat Biology and Genetic Improvement in North Yellow and Huai River Valley, Ministry of Agriculture, Crop Research Institute, Shandong Academy of Agricultural Sciences, Jinan, China

**Keywords:** quinoa-wheat reconstituted system, crispy biscuits, starch physicochemical properties, dough rheological properties, *in vitro* starch digestibility

## Abstract

Quinoa is a pseudo-cereal which has excellent nutritional and functional properties due to its high content of nutrients, such as polyphenols and flavonoids, and therefore quinoa serves as an excellent supplement to make healthy and functional foods. The present study was aimed to evaluate the quality characteristics of wheat doughs and crispy biscuits supplemented with different amount of quinoa flour. The results showed that when more wheat flour was substituted by quinoa flour, proportion of unextractable polymeric protein to the total polymeric protein (UPP%) of the reconstituted doughs decreased and the gluten network structure was destroyed at a certain substitution level. The content of B-type starch and the gelatinization temperature of the reconstituted flours increased. The storage modulus, loss modulus, development time, and stability time of the dough increased as well. Moreover, hardness and toughness of the formulated crispy biscuits significantly decreased. Analyses suggested that starch digestibility was reduced and resistant starch content increased significantly. Taken together, quinoa flour improved dough rheological properties, enhanced the textural properties, and increased resistant starch content in crispy biscuits, thus adding to high nutritional value.

## Introduction

Biscuits have become an indispensable bakery product for people worldwide due to their sensory attributes, convenience and countless varieties (1). However, biscuits are made from refined wheat flour, and thus they have unbalanced nutrients and a high glycemic index, disadvantages to patients with chronic diseases such as diabetes, obesity and hypertension (2). It is necessary to substitute part of wheat flour with other nutrition-fortifying flours to produce biscuits with optimal nutritional quality and low starch digestibility.

Quinoa (*Chenopodium quinoa* Willd.), known as a pseudo-cereal, is not only an excellent source of protein, starch, vitamins, and other basic nutrients, but also rich in functional nutrients such as polyphenols, flavonoids, and saponins compared to most cereal grains (3, 4). Despite its nutritional benefits, quinoa has a nutty taste, a different flavor from that of wheat products and its baking quality is poor (4). Therefore, quinoa alone is not a popular staple food. One of its potential usages is to incorporate into biscuit formulations as a supplement, and to improve their nutritional characteristics.

Utilization of quinoa in baked products is not popular owing to its lack of gluten protein to form viscoelastic dough. Dough properties are the dominant factors determining the baking attributes of wheat flour products (5). Rheological property of dough is usually an index of dough viscoelasticity measurement. When wheat flour is mixed with water, gluten proteins absorb water and expand to form gluten network, which confers unique viscoelastic properties on dough. However, crispy biscuits made from the reconstituted flours are expected to have the combined baking properties of wheat dough and the high nutritional properties of quinoa.

The composition and structure of gluten protein have a great role in the dough processing (6). Wheat gluten protein consists of glutenins and gliadins, and determines the viscoelastic behavior of dough. The glutenin polymers are classified into unextractable and extractable polymeric proteins (UPP and EPP) based on solubility in SDS solution (7). UPP%, as one of the commonly used indices to evaluate wheat protein quality, has a positive correlation with dough rheological properties (7, 8). Therefore, it is very promising to introduce this parameter into other non-wheat crops to analyze protein characteristics. UPP% has been used to analyze the protein quality characteristics of hulless barley-wheat reconstituted flour and UPP% of wheat-hulless barley system decreased with the increase of hulless barley proportion (9). The performance of gluten microstructure affects dough behaviors during the dough formation process (10), which can be characterized by the parameters i.e., protein area, protein junctions, and lacunarity used in protein network analysis (11, 12). However, the previous studies have rarely quantitatively analyzed the effect of quinoa supplementation on the microstructure of wheat doughs.

Starch is also a primary ingredient of dough, which is mainly responsible for pasting and thermal properties, water absorption and stability of dough structure, and consequently biscuit quality (13–15). Physicochemical properties of starch are also crucial factors affecting the quality of quinoa products (16). Starch granules with different sizes contribute differently to the dough rheological behavior, and the smaller-sized (B-type) granules improve wheat dough rheological properties (17, 18). Quinoa has a rather low apparent amylose content (4–25%) (19) but high content of super-long-chain amylopectin (20). Apparent amylose affects starch physicochemical properties, including structures, thermal and pasting properties and digestibility of starch (21). Starch is divided as rapidly digestible starch (RDS) (digested within 20 min), slowly digestible starch (SDS) (digested between 20 and 120 min) and resistant starch (RS) (digested beyond 120 min) (22) based on their digestion rate. White bread contains a high content of RDS which leads to an increase in postprandial glucose level during digestion (23). As RS can resist the decomposition of enzymes and slow release of glucose, it plays an important role in regulating blood sugar (24). Therefore, the increase in RS content in diet can contribute to nourishment and human health. Although the nutritional composition and sensory properties of quinoa-fortified bakery products have been extensively investigated (25–28), few studies have reported the relationship between the physiochemical and rheological properties of a quinoa-wheat dough system for the purpose of biscuit bakery.

In this study, quinoa and a wheat variety P13 with weak gluten were used to investigate processing quality and food functional properties of quinoa-wheat doughs and biscuits. The physicochemical properties of starch and gluten, and rheological properties of the reconstituted doughs were determined; and the physicality, texture, sensory evaluation, and starch digestibility of the biscuits were characterized. The results of this study give a new insight into analyzing the processing quality of quinoa-wheat biscuits as functional products.

## Materials and Methods

### Materials

A wheat variety, P13 with weak gluten, was selected as the source of base flour provided by Shandong Academy of Agriculture Sciences. The fine flour was acquired by milling in a Brabender Quadrumat Senior (Hackensack, United States) and then sieved (100 mesh). Quinoa flour was provided by Qinghai University with saponins removed. The samples were stored at 4^°^C for subsequent experiments.

### Determination of Basic Nutritional Components of Quinoa and Wheat Flours

A Diode Array 7,250 NIR spectrometer analyzer (Perten Instrument AB, Sweden) was used to determine moisture and ash content for the quinoa and wheat flours. Protein content was measured according to the AACC methods 46-11A. Component analyses of each flour sample were conducted in triplicate.

### Analysis of Functional Components

#### Extraction Procedure

Quinoa and wheat flour samples (0.5 g for each) were individually dissolved in 50% (v/v) ethanol (10 mL), and the mixture was extracted through reflux in 60^°^C water bath for 2 h. The total extract was obtained by repeating refluxing and stored at –20^°^C for further analyses. The sample extract was mainly used for the determination of total flavone and total polyphenol contents.

#### Total Polyphenol Content Assayed by Folin-Ciocalteau Reagent

Total polyphenol content in the two flour samples was measured using the Folin-Ciocalteau assay according to the method of Alvarez-Jubete et al. (29), applying minor changes. Sample extract (1 mL), Folin-Ciocalteau reagent (1 mL), 1 mM Na_2_CO_3_ (3 mL) and distilled water (5 mL) were added into a 10 mL tube. The mixture was placed in the dark for 15 min at 25^°^C. The absorbance of the mixture was measured at 725 nm using a UV-2100 spectrophotometer (Shanghai, China). Each sample was measured three times. A standard solution was prepared using gallic acid and a calibration curve was obtained using a range of concentrations from 0.01 to 0.06 mg/mL. The results were expressed as gallic acid equivalent (GAE) in mg/g of dry-weight basis. Gallic acid was purchased from Sangon Biotech (Shanghai) Co., Ltd. (Shanghai, China).

#### Total Flavone Content Assay by Aluminum Nitrate Colorimetric Method

Total flavone content in the two samples was measured using the aluminum nitrate colorimetric method (30) with minor modifications. Sample extract (1.0 mL), 0.72 M NaNO_2_ (0.15 mL), 0.27 M Al_2_ (NO_3_)_3_ (0.15 mL), 1 M NaOH (2.0 mL) and 60% (v/v) ethanol (1.7 mL) were sequentially added into a 5 mL tube. The mixture was incubated for 15 min at 25^°^C. Absorbance of the mixture was measured at 517 nm using a UV-2100 spectrophotometer (Shanghai, China). Each sample was measured three times. A standard solution was prepared using rutin and a calibration curve was obtained with a range of concentrations from 0.75 to 75 μg/mL. Total flavone content was expressed as mg rutin equivalent (RE)/g of sample. Rutin was purchased from Sangon Biotech (Shanghai) Co., Ltd. (Shanghai, China).

### Preparation of Flour Samples

The quinoa flour was added to substitute for wheat flour at five ratios: 10, 15, 20, 25, and 30% (w/w), and the reconstituted flours were designated as Q10, Q15, Q20, Q25, and Q30, with wheat base flour (Q0) as control. The reconstituted flours were kept at 4^°^C for subsequent experiments.

### Analysis of Starch Physicochemical Properties

#### Isolation of Starch

Wheat starch was isolated from flour following the method reported by Zi et al. (18) with modifications. Wheat starch was extracted with 75% (v/v) ethanol and dried in an oven at 55^°^C overnight, ground into powder using a blender and sieved through a 200-mesh sieve. Starch in reconstituted flours was also isolated according to the above method.

Starch was isolated from quinoa flour as described by Contreras-Jiménez et al. (31), with minor modifications. The mixture of quinoa flour and distilled water (1:3, w/v) was maintained at 25^°^C for 1 d. The mixture was sieved (80 mesh), rewashed with 75% (v/v) ethanol and centrifuged at 1,503 × *g* for 20 min to remove the supernatant. The gray layer on the precipitated surface was scraped off and the remaining pellet (quinoa starch) at the bottom were rewashed with 75% (v/v) ethanol and centrifuged. The quinoa starch was obtained by washing the samples for 3–5 times and dried at 60^°^C for 1 day. The starch sample was ground into fine powder, sifted (200-mesh) and stored at 4^°^C for subsequent operations.

#### Measurement of Apparent Amylose Content

Apparent amylose content of individual quinoa and wheat starch sample was measured with an Amylose/Amylopectin Assay Kit (Megazyme International, Ireland Ltd., Bray, Ireland) in triplicate.

#### Analysis of Size Distribution of Starch Granules

The number of different sized starch granules was determined using a laser diffraction instrument (Microtrac S3500, United States) in triplicate according to the method of Yu et al. (32). The raw data was exported to calculate starch granule number distribution using Microsoft Excel 2019 software.

#### Thermal Properties

Thermal properties of starch samples were analyzed using a simultaneous thermogravimetric analyzer (STA 7200 RV, HITACHI, Japan) according to an established method (13). Starch sample (3.0 mg) was placed in an aluminum crucible (Ø 5.2 × 2.5 mm) on a sample holder. A blank pan was placed on the reference side sample holder. The experiment was carried out in a dynamic nitrogen environment in the range of 30–300^°^C, with temperature increasing at a rate of 10^°^C/min. Four parameters were determined by TA 7000 standard analysis software: onset temperature (T_0_), peak of gelatinization temperature (T_*p*_), conclusion temperature (T_*c*_) and gelatinization enthalpy (ΔH_gel_). The measurement was repeated thrice for each sample.

### Determination of UPP% by Size-Exclusion High Performance Liquid Chromatography

UPP and EPP in the reconstituted flours were extracted referring to an established method (33). UPP% in the glutenin fraction was determined as the ratio between peak area of UPP and total peak area of UPP and EPP on an size-exclusion high performance liquid chromatography (SE-HPLC) system (Infinity 1260, Agilent, United States). Each sample was measured with two replicates and the average values were calculated.

### Observation of Dough Microstructure

Microstructure of the reconstituted dough samples was visualized by confocal laser scanning microscopy (Olympus, Tokyo, Japan) as described by Bernklau et al. (11). Rhodamine B (0.01 mg/mL) was used for dying the protein. The dough samples were prepared in duplicate. Five independent images (512 × 512 pixel, 423.108 × 423.108 μm) were obtained for each dough sample. The AngioTool 64 version 0.6a (National Cancer Institute, Maryland, United States) was used to qualitatively analyze each image with the three parameters: protein area, protein junctions and lacunarity.

### Determination of Dough Rheological Properties

A rheometer (RotoViscol, HAAKE, Germany) was used to determine the small amplitude oscillatory shear measurements of the reconstituted doughs, as described by Wang et al. (34). Storage modulus (G′), loss modulus (G″) and Tan δ (G″/G′) were determined in duplicate.

Mixing properties of the reconstituted doughs were determined using a Mixolab instrument (Mixolab2, Chopin, France) following the method reported by Niu et al. (35). Mixolab analysis was carried out in the process of heating and mixing, which determined the characteristics of gluten strength and starch viscosity of the dough. The dough mixing behaviors were recorded with the standard “Chopin+” protocol. Eleven dough mixing parameters were determined and calculated on the dough processing curve: water absorption (WA), dough development time (DDT), dough stability time (DS), dough development (C1), protein weakening (C2), starch peak viscosity (C3), trough viscosity (C4), final viscosity (C5), protein weakening (%), starch breakdown (C3-C4) and starch retrogradation (C5-C4). Two replications were conducted for each sample.

### Preparation of the Formulated Crispy Biscuits and Analysis of Functional Components

Formulated crispy biscuits were made according to AACC method 10–50D with minor modifications. The dough samples were prepared by mixing 100 g reconstituted flour, 15 g shortening, 28.5 g erythritol, 2 g cream, 0.3 g salt, 0.07 g sodium bicarbonate, 0.3 g ammonium bicarbonate, 16 g egg and a proper amount of water (acquired from the Mixolab data: Q0: 10.22 mL; Q20: 10.54 mL; Q25: 10.61 mL; Q30: 10.60 mL) at 22^°^C, followed by resting for 10 min. Dough was pressed to a thickness of 5.0 mm with a rolling pin, cut with a 50 mm diameter die and baked in an oven at 200^°^C for 9 min. Two batches of biscuit replicas were prepared and cooled to 25^°^C for further analyses. Shortening, erythritol, cream, salt and egg were purchased from local supermarket. Additionally, “Observation of dough microstructure” and “Determination of dough rheological properties” were made using dough prepared as described in “Preparation of the formulated crispy biscuits.”

The contents of total polyphenol and total flavone in biscuit samples were determined according to the method of quinoa and wheat flours in triplicate.

### Evaluation of the Crispy Biscuit Baking Performance

#### Physical Properties Measurement

Biscuit samples were weighted using a laboratory balance (BSA223S, Sartorius, Germany) and their diameter and thickness were measured using a ruler. Two batches of biscuit replicas were measured and the mean was calculated.

#### Texture Measurement

Hardness and toughness of the formulated biscuits were measured using TVT Texture Analyzer (TVT 6700, Perten, Sweden) (28). The biscuit sample was placed stably on the center of the base on the testing table. The probe (P-BP70A) and the base (R-TPBR) were selected, the compression height was set to 15.00 mm; the pretest speed was 2.5 mm/s; the test speed was 2.0 mm/s and the posttest speed was 10.0 mm/s. The values are presented as the average of two measurements.

#### Sensory Evaluation

Sensory characteristics of crispy biscuit samples with different percentages were evaluated using untrained consumers (22–55 years old). The panelists were asked to evaluate the color, shape, structure, flavor and taste of samples and overall acceptability. All parameters were compared with control sample without quinoa addition (Q0). The ratings were on the 9-point hedonic scale ranging from 9 (like extremely) to 1 (dislike extremely) for each characteristic.

#### In vitro Starch Digestion Assay

Referring to Toutounji et al. (36), *in vitro* digestion of the formulated biscuits was analyzed by sequentially mixing biscuit sample (5 g), sodium acetate solution (40 mL, 0.2 M, pH 6.0) and working enzyme solution (5 mL, 0.66 mL 200 U/mL porcine pancreatin + 1 mL 100 U/mL amyloglucosidase, added up to 50 mL with buffer) in a test tube. The mixture (after being vortexed) was kept in a shaker at 37^°^C while rotating at 230 rpm for 2 h. The aliquot samples (0.2 mL at each time) of the mixture were collected at 0, 20 and 120 min. The test tubes were immediately heated in 100^°^C bath for 8 min to denature enzymes and centrifuged at 15,871 × *g* for 10 min. The glucose present in the supernatant was measured at each time point using a D-Glucose Assay kit (GOPOD method, K-GLUC 09/14, Megazyme International, Ireland Ltd., Bray, Ireland) and a UV-2100 spectrophotometer (Shanghai, China). Amyloglucosidase (Aspergillus niger, 100,000 U/mL) was purchased from Macklin Biochemical Co., Ltd. (Shanghai, China), and porcine pancreatin (4 USP) was purchased from Sigma-Aldrich Ltd. (St. Louis, MO, United States).

The relative proportions of RDS, SDS, and RS were calculated (4). The formulas are as follows:


$$RDS\left(\%\right)=\left(G_{20}-G_{0}\right)\times 0.9\times 100$$

$$SDS\left(\%\right)=\left(G_{120}-G_{20}\right)\times 0.9\times 100$$

RS(%)=[(TS-RDS-SDS)÷TS]×100

where G_0_, G_20_, and G_120_ represent the content of glucose (%) released after 0, 20, and 120 min, respectively. TS is total starch content of the sample, and 0.9 is the conversion factor for glucose converted to anhydroglucose (the starch monomer unit).

### Statistical Analysis

The resultant data were processed by SPSS software (SPSS Inc., version 22.0 United States). Differences between components of quinoa and wheat flour were assessed by Student’s *t*-test (*P* ≤ 0.05). The reconstituted flours were analyzed by one-way analysis of variance (ANOVA) and the differences among flour samples were evaluated by least significant difference (LSD) (*P* ≤ 0.05).

## Results and Discussion

### Compositional Characteristics of Quinoa and Wheat Flours

Ingredients of quinoa and wheat flours are shown in Table 1. The results shows that moisture and apparent amylose content of quinoa flour were lower than those of wheat flour. There was significant difference in protein content between quinoa and wheat flours. Quinoa has been used as a supplement to improve nutritional quality and to fortify food products (5). Previous studies have shown that the protein content of quinoa is generally higher than that of common grains (i.e., wheat, corn and barley) (3, 4). Ash content of quinoa flour is higher than that of wheat flour, which may be attributed to crop mineral content (31). The results of this study on compositional characteristics of quinoa and wheat flours are comparable to the previous results (31, 37). As for functional components, quinoa flour contains significantly more total polyphenol and total flavone than wheat flour does. It is generally accepted that polyphenols have beneficial effects on health and polyphenols are the most abundant antioxidants that can scavenge free radicals such as 2,2-diphenyl-1-picrylhydrazyl (DPPH) (38). Therefore, the above result indicates that quinoa has stronger antioxidant capacity than wheat does. Referred to in the earlier studies, total polyphenol content in quinoa ranged from 1.48 to 5.18 mgGAE/g (25, 39). Total flavone content in two quinoa varieties (Salcedo and Altiplano) was 8.69 and 9.14 mg RE/g, respectively (40). The results of the current study were different from the previous results: total polyphenol content in quinoa was relatively high, while total flavone content relatively low, which can be attributed to many factors, such as variations among crop varieties, environmental conditions and cultivation methods. In addition, different polyphenol and flavone extraction methods had significant effects on the results (29). Given that the current results demonstrate quinoa has higher nutritional values than wheat does, the study of the quinoa-wheat flour for biscuit baking is essential.

**TABLE 1:** Compositional characteristics of quinoa and wheat flours.

Sample	Moisture (%)	Protein content (%)	Ash content (%)	Apparent amylose content (%)	Total polyphenol content (mgGAE/g)	Total flavone content (mgRE/g)
Quinoa flour	9.64 ± 0.43b	16.81 ± 1.75a	2.17 ± 0.04a	18.74 ± 1.63b	8.76 ± 0.71a	3.00 ± 0.52a
Wheat flour	11.28 ± 0.07a	10.68 ± 0.17b	0.93 ± 0.01b	28.36 ± 1.56a	3.88 ± 0.01b	0.27 ± 0.01b

*Values followed by different letters in the same column are significantly different (P < 0.05).*

### Analyses of Particle Size Distribution and Thermal Properties of Starch

Different methods can be applied to extract starch from quinoa and wheat, since wheat dough can be separated into starch suspension and gluten by hand washing while quinoa dough can’t. The results of particle size distribution of starch showed that the content of B-type granules increased obviously when quinoa flour was added (Table 2), which is consistent with the previous findings that most of the quinoa starch granules are composed of small granules (diameter from 1 to 3 μm) (19, 20). Generally, A-type starch granules have higher amylose content (41, 42). As shown in Table 1, apparent amylose content of quinoa flour is lower than that of wheat flour, which can be explained that quinoa flour is composed of small granules. Given that more B-type granules can improve the dough rheological properties (9, 17), the reconstituted flours are expected to show better rheological behavior.

**TABLE 2:** B-type starch granule content and thermal properties of starch from the reconstituted flours.

Sample	B-type starch granule content (%)	Thermal property			
		T_0_ (^°^C)	T_p_ (^°^C)	T_c_ (^°^C)	ΔH_gel_ (J/g)
Q0	68.85 ± 2.45b	31.75 ± 0.21e	64.15 ± 0.07c	74.70 ± 0.00c	86.05 ± 0.78a
Q10	68.92 ± 2.22b	31.80 ± 0.14e	63.55 ± 0.92d	73.05 ± 0.21d	82.90 ± 0.00b
Q15	69.95 ± 0.14b	32.05 ± 0.07d	63.95 ± 0.07c	75.15 ± 0.78c	81.05 ± 0.21c
Q20	71.82 ± 1.33b	32.40 ± 0.14c	64.15 ± 0.07c	77.50 ± 0.71b	78.10 ± 2.55d
Q25	72.91 ± 2.15b	32.90 ± 0.00b	65.40 ± 0.00b	77.65 ± 0.21b	74.55 ± 0.78e
Q30	79.07 ± 1.54a	33.50 ± 0.28a	68.45 ± 0.35a	78.85 ± 0.21a	77.75 ± 0.64d

*Values followed by different letters in the same column are significantly different (P < 0.05). T_0_, onset temperature; T_p_, peak of gelatinization temperature; T_c_, conclusion temperature; ΔH_gel_, gelatinization enthalpy.*

The gelatinization properties for wheat starch and quinoa-wheat starches were determined by STA, and the results are shown in Table 2. With the increasing proportion of quinoa flour, T_0_, T_p_, and T_c_ increase significantly, whereas ΔH_gel_ decreases (Table 2). A similar phenomenon has been observed when different proportions of quinoa flour were added to wheat flour and potato starch (27). Thermal properties of starch were closely related to the molecular architecture of crystalline regions of amylopectin (43). Since the long unit chains in amylopectin can allow more flexibility of double helices, which makes the crystalline region of amylopectin more orderly (13, 44), the high content of super-long-chain amylopectin in quinoa may be responsible for increasing gelatinization temperature of starch in the reconstituted flour system. Starch with higher gelatinization temperature possesses improved molecular order and crystallinity, which requires more energy for gelatinization, and consequently exhibits delayed gelatinization. Different particle size distribution may also cause differences in starch thermal properties. Compared with A granules, B granules showed higher T_0_, T_p_, and T_c_, but lower ΔH_gel_. With the increase of B/A ratio, gelatinization temperature of mixed starches increased while the ΔH_gel_ decreased (45, 46). Taken together, it can be inferred that the increase of gelatinization temperature and the decrease of ΔH_gel_ may be ascribable to the increase of B-type content in the reconstituted systems.

### Analyses of UPP% and Gluten Network Structure of Reconstituted Flours

UPP% is often used as an index to evaluate the wheat quality (6, 9), and in this study it is introduced to evaluate the quinoa quality. Consequently, the UPP% of quinoa-wheat flours was explored and its effect on the dough strength investigated. SE-HPLC measurements showed that the UPP% of the reconstituted flours decreases by 6.27–16.14% compared with Q0, and the graph shows an S-shaped downward trend (Supplementary Figure 1A). The results showed addition of quinoa flour significantly lower UPP%, indicating quinoa weakened wheat dough strength. Accordingly, the microstructure of dough was quantitatively analyzed by AngioTool to explore its internal mechanism (Supplementary Figure 1). Referred to by Bernklau et al. (11), denser protein cross-linkage contributes to greater stability of gluten and dough, which can be characterized by protein area and protein junctions. Larger protein area and more protein junctions indicates that the gluten protein network structure is more closely connected. The reconstituted doughs except Q10 showed less protein area and fewer protein junctions than Q0 did (Supplementary Figures 1B,C), indicating that when quinoa flour was added into wheat flour, the gluten network structure in the dough system deteriorated. The protein area and protein junctions of Q10 increased slightly, without significant difference, compared to those of Q0. Lacunarity reflects the regularity of voids in the network structure and a higher value indicates weaker dough strength (9, 47). With the addition of quinoa flour, lacunarity became larger, indicating the reconstituted doughs became weaker (Supplementary Figure 1D).

Combined with the result of UPP%, it can be concluded that the gluten protein quality of the reconstituted doughs became worse with the addition of quinoa flour. The results can be explained by the fact that quinoa does not contain gluten, so that the protein of quinoa cannot connect with wheat gluten to form stable and complete gluten network structure (4). Since the microstructure of the reconstituted doughs was disrupted, the “weakened” doughs are expected to make different crispy biscuits.

### Rheology

**FIGURE 1:**
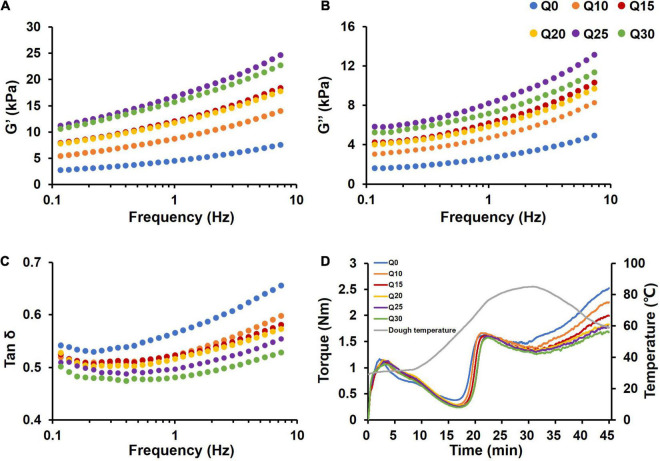
Rheological properties of wheat and quinoa reconstituted doughs. **(A)** Storage modulus (G′). **(B)** Loss modulus (G″). **(C)** Tan δ. **(D)** The mixing profiles of doughs. The data were obtained from two replicates of each sample.

It is generally recognized that the rheological properties of dough are related to the quality of the final product in some way (48). A rheometer was used to investigate the rheological behavior of dough. The effect of quinoa flour on the rheological properties of wheat dough was analyzed (Figure 1). G′ and G″ represent solid-like and liquid-like characters of the test dough samples, respectively (49). When frequency was in the range of 0.1–10 Hz, G′ and G″ increased in a frequency-dependent manner. G′ was always higher than G″, which led to tan δ < 1 (Figures 1A–C), indicating predominance of a solid-like character for the reconstituted dough samples. The reconstituted doughs exhibited higher G′ and G″, compared with Q0. Tan δ decreased with an increased proportion of quinoa flour in dough samples, indicating quinoa flour improved dough viscoelasticity.

It has been generally accepted that UPP% is positively correlated with the dough rheological properties (6, 50). Protein polymers should have disintegrated as inclusion of quinoa flour diluted the gluten network. However, in this study, the rheological properties of the dough were improved. This may be attributed to that the starch of quinoa contains more small-sized granules, which combine more closely with gluten network (9, 51). The improved effect of quinoa starch may offset the decreased effect of the diluted gluten. In the current study, the increased content of smaller-sized starch in the dough system affected physicochemical and structural properties of the wheat dough because the quinoa starch granules embedded in the gluten network structure improve the stability and the rheological properties of the dough.

The dough rheological properties were further analyzed using a Mixolab instrument. Mixolab mixing curves suggested that there was a significant difference in torque among the tested samples at the dough formation stage (0–8 min), and the time from the initial to C1 was significantly lengthened with more quinoa flour added (Figure 1D). The Mixolab parameters can reflect the effect of the quinoa on gluten strength and starch pasting properties (Table 3). WA of flour is affected by multiple factors including protein and starch (43). The presence of quinoa significantly increased WA of the flours in a linear manner. Given that B-type starch granules with larger surface area can combine more water molecules, and thus increase water absorption of dough (18, 52), we attribute increased WA to inclusion of B-type starch granules in the reconstituted flours. It is noteworthy in this study that DDT and DS of the reconstituted doughs showed a stepwise increase. Inconsistent with the previous studies (30, 53), the absence of gluten in quinoa destroyed the gluten network structure, but prolonged DDT and DS, which can be explained by the assumption that under mechanical force, the protein-protein and protein-starch interactions between quinoa and wheat occurred. Also, high water absorption resulted in more available water in the reconstituted doughs thereby improving the homogeneity and stability of the gluten-starch matrix in the dough system (54). Since the reconstituted doughs showed better performance in the dough development than the control Q0 did, it is clear that quinoa flour at certain substitution levels improved the mixing properties of wheat dough.

**TABLE 3:** Effect of quinoa content on the mixing properties of wheat dough.

Sample	WA (%)	DDT (min)	DS (min)	C1 (Nm)	C2 (Nm)	C3 (Nm)	C4 (Nm)	C5 (Nm)	Protein weakening (%)	Breakdown (C3-C4) (Nm)	Setback (C5-C4) (Nm)
Q0	65.85 ± 0.07e	2.19 ± 0.05e	1.95 ± 0.21d	1.17 ± 0.06a	0.38 ± 0.04a	1.63 ± 0.00b	1.45 ± 0.05a	2.52 ± 0.00a	35.10 ± 0.01d	0.17 ± 0.05c	1.07 ± 0.05a
Q10	66.30 ± 0.14d	2.81 ± 0.11d	2.85 ± 0.18c	1.15 ± 0.04ab	0.29 ± 0.01b	1.66 ± 0.02a	1.36 ± 0.02b	2.25 ± 0.01b	50.80 ± 0.01c	0.30 ± 0.00ab	0.89 ± 0.01b
Q15	67.15 ± 0.21c	3.08 ± 0.08c	3.01 ± 0.01c	1.14 ± 0.03b	0.27 ± 0.00c	1.61 ± 0.00b	1.32 ± 0.01c	1.99 ± 0.01c	54.00 ± 0.00b	0.29 ± 0.01ab	0.67 ± 0.00c
Q20	67.90 ± 0.00b	3.12 ± 0.12c	3.62 ± 0.05b	1.15 ± 0.03b	0.28 ± 0.01bc	1.61 ± 0.00b	1.32 ± 0.01c	1.83 ± 0.00d	58.90 ± 0.01a	0.29 ± 0.01b	0.50 ± 0.01d
Q25	68.35 ± 0.21a	3.26 ± 0.08b	3.79 ± 0.12b	1.07 ± 0.00c	0.25 ± 0.04d	1.58 ± 0.01c	1.28 ± 0.02d	1.74 ± 0.02e	55.80 ± 0.01b	0.31 ± 0.01a	0.46 ± 0.00e
Q30	68.30 ± 0.28a	3.57 ± 0.19a	4.54 ± 0.16a	1.08 ± 0.02c	0.25 ± 0.08d	1.58 ± 0.10c	1.28 ± 0.02d	1.69 ± 0.01e	54.50 ± 0.00b	0.30 ± 0.08ab	0.41 ± 0.01f

*Values followed by different letters in the same column are significantly different (P < 0.05). WA, water absorption; DDT, dough development time; DS, dough stability time; C1, dough development; C2, protein weakening; C3, starch peak viscosity; C4, trough viscosity; C5, final viscosity.*

When the temperature began to rise, the dough entered the protein weakening stage (C2) (Figure 1D). Torque began to drop because of protein denaturation, and continued until starch gelatinization began (30). Protein weakening (%) is described as difference between torque at 8 min and at C2. The protein weakening degree of the reconstituted doughs increased more significantly than that of wheat dough, indicating that quinoa increased protein weakening (Table 3), which was consistent with the finding about UPP% (Supplementary Figure 1). Similar results were reported by Gujral et al. (53), when hulless barley bran was supplemented to refined wheat flour for chapatti making. This is because the reconstituted doughs contained lower levels of gluten and weakened microstructure, which caused higher protein weakening degree and decreased dough consistence at C2.

As the temperature continued to rise, the dough entered the starch-dominating stage in the mixing process. At C3, starch peak viscosity was determined, which is a pivotal index to evaluate the end-use properties of food products (14). During the stage, torque for Q0 increased greatest (Figure 1D). C3 torque of the reconstituted doughs decreased slightly, indicating lower peak viscosity for quinoa than for wheat flour (Table 3). This might be attributed to mild variations in water absorption and swelling of small-sized starch granules. Because of the smaller volumes, the B-type starch granules have relatively smaller swelling expansion, which is responsible for lower peak viscosity (41). Besides, other factors, such as the contents of dietary fiber and polyphenols rather than of starch, have been reported to affect pasting properties, which cannot be negligible (20, 30). These components competed with starch granules for water, resulting in a decrease in the available water combined with starch and thus affected the pasting properties of starch.

When the dough temperature continued to rise to the maximum, torque continuously decreased until trough viscosity occurred (during C4). Breakdown is one of parameters that indicates paste stability (55). The breakdown of reconstituted doughs ranged from 0.29 to 0.31 Nm, which is higher than that of Q0 (0.17 Nm), indicating the reconstituted doughs had lower hot-paste stability (Table 3). Hot-paste stability is closely related to the exudation of amylose after the starch granule ruptured (56). The difference in structure between quinoa and wheat starch may be more likely to accelerate the leaching of amylose after starch granules fall off, resulting in poor stability.

During the dough cooling stage, torque increased owing to the recrystallization of gelatinized starch granules (53). With the higher ratio of quinoa flour, final viscosity of the reconstituted doughs decreased significantly (Table 3). Setback, the difference of torque between C5 and C4, is defined as the process of recrystallization of starch during cooling, which is related to retrogradation tendency of starch (27). With the addition of quinoa flour, setback values showed a downward trend. Shelf life is particularly important for biscuits. Considering the contribution of starch retrogradation to biscuits staling, a decrease in setback value for the reconstituted doughs indicated that the retrogradability of the reconstituted doughs decreased and thereby the shelf-life of biscuits can be extended (21, 57). The possible reason might be that higher polyphenol content in quinoa with higher anti-oxidation ability can inhibit the retrogradation of starch (30). In addition, amylose content is also one of the important factors affecting starch retrogradation (20). Amylopectin has many branches and complex structure, which has a large space barrier and will slow retrogradation in solution; while amylose in solution has little space barrier and is ready to facilitate retrogradation. Therefore, although quinoa flour increased the torque value at protein weakening in the mixing process, it strongly improved starch retrogradability, showing that quinoa starch impacts mixing properties of wheat dough greatly.

**FIGURE 2:**
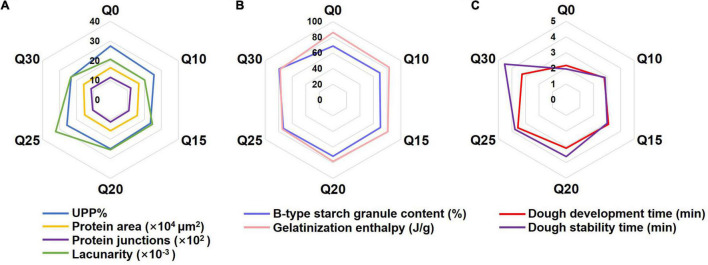
The radar graphs of quality characteristics of wheat and quinoa reconstituted doughs. **(A)** Comparison of UPP%, protein area, protein junctions and lacunarity in wheat and quinoa reconstituted doughs. **(B)** Comparison of B-type starch granule content and gelatinization enthalpy in wheat and quinoa reconstituted doughs. **(C)** Comparison of dough development time and dough stability time in wheat and quinoa reconstituted doughs.

The radar graphs were constructed to visualize the comparison among quality characteristics of doughs made from different flours with their components at different levels. Three wheat doughs supplemented with quinoa flours at three ratios (Q20, Q25, and Q30) had better overall scores than Q0 (Figure 2), which indicates the three formulations can fulfill the purpose of functional biscuit baking.

### Physical Properties, Texture Profile, and Sensory Evaluation of Crispy Biscuits

Physical property analyses showed that quinoa flours at different substitution ratios had different effects on thickness and weight of biscuits, and the effect was more evident when the quinoa content increased (Table 4). Textural properties are important for biscuit quality (15). The analyses of texture properties of the four crispy biscuits showed that hardness and toughness of Q0 was greater than those of Q20, Q25, and Q30 (Table 4), which is consistent with the previous report (2). Hardness and toughness are considered as important parameters to evaluate biscuit quality (15). Differences in moisture content of the dough also contribute to differences in biscuits crispiness (58). When quinoa flour was added to wheat flour, WA increased, resulting in an increase in the moisture content of dough (Table 3) and a decrease in hardness of the formulated crispy biscuits. Lower hardness and greater toughness are favorable attributes of the crispy biscuits. The above results showed that among the four biscuit samples, Q25 had superior textural attributes.

**TABLE 4:** Effect of quinoa content on the physical property measurements, textural properties, sensory evaluation, and *in vitro* starch digestibility of crispy biscuits.

	Parameter	Q0	Q20	Q25	Q30
Physical property	Diameter (cm)	5.68 ± 0.02a	5.65 ± 0.06a	5.65 ± 0.11a	5.63 ± 0.04a
	Thickness (mm)	9.96 ± 0.76b	10.52 ± 0.33b	10.85 ± 0.32ab	11.49 ± 0.43a
	Weight (g)	15.93 ± 0.56b	18.47 ± 0.66a	19.15 ± 1.05a	19.29 ± 0.93a
Textural property	Hardness	72.83 ± 1.64a	73.99 ± 9.49a	50.16 ± 8.33b	57.34 ± 4.52b
	Toughness	10.01 ± 0.01a	5.48 ± 0.49bc	6.66 ± 1.56b	5.34 ± 0.76c
Sensory evaluation	Color	8.10 ± 0.26a	7.74 ± 0.10b	7.38 ± 0.20c	6.66 ± 0.12d
	Shape	8.10 ± 0.26a	7.74 ± 0.17b	7.56 ± 0.19b	7.02 ± 0.30c
	Structure	7.20 ± 0.00c	7.56 ± 0.00a	7.38 ± 0.20b	7.20 ± 0.00c
	Flavor	8.10 ± 0.23a	7.38 ± 0.25b	7.02 ± 0.25b	6.12 ± 0.41c
	Overall acceptability	8.00 ± 0.23a	8.12 ± 0.11a	7.77 ± 0.20a	7.10 ± 0.16a
*In vitro* starch digestibility	RDS (%)	60.80 ± 0.30a	59.21 ± 1.09b	56.70 ± 0.13c	51.47 ± 0.07d
	SDS (%)	1.86 ± 0.53c	1.15 ± 0.03d	3.26 ± 0.63b	4.95 ± 0.13a
	RS (%)	37.34 ± 0.82c	39.64 ± 0.03b	40.04 ± 0.46b	43.58 ± 0.00a

*Values followed by different letters in the same row are significantly different (P < 0.05). RDS, rapidly digested starch; SDS, slowly digested starch; RS, resistant starch.*

The results of hedonic sensory scores for crispy biscuits were given in Table 4. In terms of color and shape, the scores of Q0, Q20, Q25, and Q30 decreased sequentially. It can also be seen from Supplementary Figure 2 that the appearance and color of Q20 were better than that of Q25 and Q30. The biscuits displayed varied colors from light to dark brown (Supplementary Figure 2), which may be attributed to browning because of Maillard or caramelization reactions. This agrees with the report that temperature and quinoa flour instead of wheat flour affect the browning of biscuits (59). Additionally, the deeper browning of quinoa samples reported in previous studies is due to high levels of polyphenols and ash (2), which is consistent with the results of this study. The inclusion of quinoa in crispy biscuits resulted in increased scores for structure compared to Q0. However, supplement of quinoa in crispy biscuits reduced scores for flavor. In spite of undesirable results in terms of color, shape, and flavor, Q20 ensured satisfactory overall consumer acceptability of formulated crispy biscuit. Although the use of quinoa flour resulted in a slight decrease in the overall acceptability values, all formulated crispy biscuits including Q30, which was substituted with most quinoa, remained within the range that consumers appreciate with the overall rating acceptable.

### *In vitro* Starch Digestibility

**FIGURE 3:**
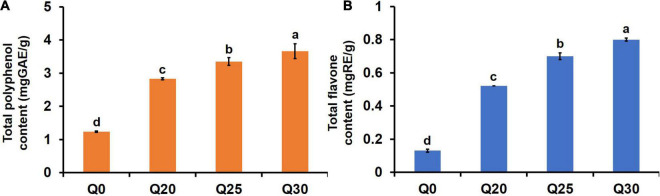
Effects of quinoa addition on functional components in the flour crispy biscuit samples. **(A)** The total polyphenol content in the crispy biscuit samples. **(B)** The total flavone content in the crispy biscuit samples. The letters indicate significant differences (*P* < 0.05).

Analysis of *in vitro* starch digestibility of the crispy biscuits shows that RDS, SDS, and RS content of Q0 are 60.80, 1.86, and 37.34%, respectively (Table 4). With the increased inclusion of quinoa flour, the digestion rate of crispy biscuit starch significantly dropped, which was confirmed by the fact that RS increased and RDS and SDS decreased. For patients with type 2 diabetes, obesity, or cardiovascular disease, foods with lower RDS content, higher SDS, and RS contents are healthier (24). Consequently, the decreased RDS and increased RS suggested that crispy biscuits supplemented with quinoa flour should be beneficial, since carbohydrates were digested slowly *in vivo*, and thereby prevented sharp increase of postprandial blood glucose. Amylase was considered to be a key factor in determining starch digestibility (14). Starch digestibility of the crispy biscuits was decreased with the increase of quinoa flour. Wang et al. (60) reported that in the process of digestion, A-type wheat starch gelatinized largely, while B-type wheat starch and quinoa starch granules were wrapped in a protein-sugar-oil film after baking, forming a natural barrier, its digestion rate was slower. In this study, due to the presence of quinoa starch, the content of small starch granules in the reconstituted flour increased, which reduced the starch digestibility. Additionally, reduced starch digestibility may also be attributed to higher phenolic and flavonoid contents restricting the hydrolysis of enzyme (30). As shown in Figure 3, the contents of total polyphenol and total flavone of Q20, Q25, and Q30 were significantly higher than those of Q0, and increased with the increase of quinoa content. Therefore, this may also be one of the reasons for the decline in digestibility. In a word, quinoa can greatly reduce starch digestibility and increase the RS content of biscuits, indicating that quinoa can effectively improve the functional attributes of crispy biscuits.

### The Relationship Between Biscuit Properties and Gluten Composition, Starch and Rheological Properties of Quinoa-Wheat Reconstituted System

**FIGURE 4:**
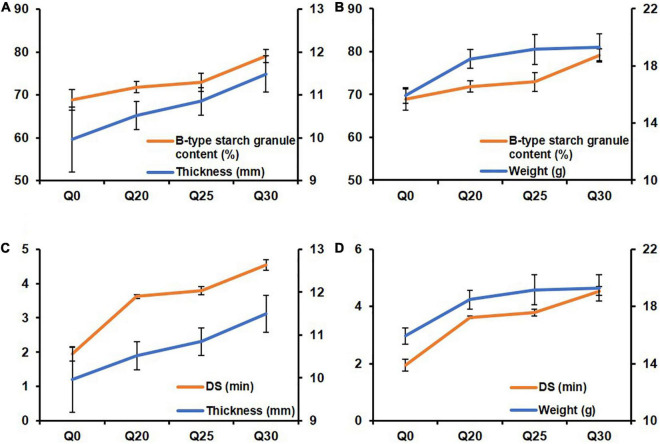
The line charts exhibiting relationship between biscuit properties and starch and rheological properties of quinoa-wheat reconstituted systems. **(A)** The relationship between B-type starch granule content and crispy biscuit thickness. **(B)** The relationship between B-type starch granule content and crispy biscuit weight. **(C)** The relationship between dough stability (DS) and crispy biscuit thickness. **(D)** The relationship between dough stability (DS) and crispy biscuit weight.

To further explore the relationship between the biscuit properties and gluten, starch and rheological properties of the quinoa-wheat reconstituted system, the line graphs were drawn for comparison. The results showed that B-type starch granule content and DS had the same change trend as biscuit thickness and weight with the increase proportion of quinoa flour (Figure 4), which is consistent with previous report (61). The possible reason for this trend is that more small starch granules are filled in the gluten network structure. During the dough formation process, the broken network structure is reorganized, thereby improving the dough stability. High-quality dough properties are conducive to volume expansion (62), resulting in increased biscuit thickness and weight.

However, there was no significant relationship between gluten properties and biscuit properties, nor between properties of starch and dough rheology and hardness and toughness of biscuit (Supplementary Figure 3), which is consistent with the previous study demonstrating no significant correlation between starch structure and biscuit hardness and fracturability (63). Obviously, there are many factors affecting the quality properties of biscuits, e.g., damage starch, pentosans (64), fiber (65), and other components in quinoa or wheat, which also requires further research.

## Conclusion

This study explored the effect of quinoa flour on physiochemical and mixing properties of wheat dough. Moreover, the physical, textural and digestion properties of the formulated crispy biscuits were analyzed. The results of the study showed that although added quinoa flour significantly disrupted the gluten network structure, it increased B-type granule content and gelatinization temperature of doughs. Interestingly, rheological properties of the reconstituted doughs were improved and dough aging was reduced. In addition, quinoa flour significantly improved the textural and digestibility properties of crispy biscuits. The contents of RDS and SDS were significantly reduced, while that of RS was significantly increased, indicating that the formulated biscuits were more beneficial to patients with special diseases (e.g., diabetes). On the whole, quinoa flour at certain levels had a positive effect on biscuit quality: not only improved rheological properties but also yielded promising results on healthy food.

## Data Availability Statement

The raw data supporting the conclusions of this article will be made available by the authors, without undue reservation.

## Author Contributions

YM: investigation, formal analysis, and writing—original draft, review, and editing. DW: methodology, data curation, writing—review, and editing. LG: investigation and data curation. YY, XY, and ZW: resources. KW, XC, and XG: supervision, project administration and writing—review, and editing. All authors contributed to the article and approved the submitted version.

## Conflict of Interest

The authors declare that the research was conducted in the absence of any commercial or financial relationships that could be construed as a potential conflict of interest.

## Publisher’s Note

All claims expressed in this article are solely those of the authors and do not necessarily represent those of their affiliated organizations, or those of the publisher, the editors and the reviewers. Any product that may be evaluated in this article, or claim that may be made by its manufacturer, is not guaranteed or endorsed by the publisher.

## Supplementary Material

Supplementary Figure 1 Gluten protein composition and microstructure of wheat and quinoa reconstituted flours and doughs. **(A)** The UPP% of wheat and quinoa reconstituted flours. **(B–D)** Microstructure indicated by the protein area, protein junctions and lacunarity of the wheat and quinoa reconstituted doughs. The data were derived from five captures of each sample and presented as mean value ± standard deviation.

Supplementary Figure 2 Image of formulated crispy biscuits appearances and textures with the scale bar of 1 cm.

Supplementary Figure 3 The line charts exhibiting unobvious relationship between biscuit properties and gluten, starch and rheological properties of quinoa-wheat reconstituted systems.
