# Supplementary figures and images for: Effects of Quinoa Flour on Wheat Dough Quality, Baking Quality, and in vitro Starch Digestibility of the Crispy Biscuits
Source: Front Nutr. 2022 Apr 13;9:846808. doi: 10.3389/fnut.2022.846808 (PMC9043647; doi:10.3389/fnut.2022.846808)

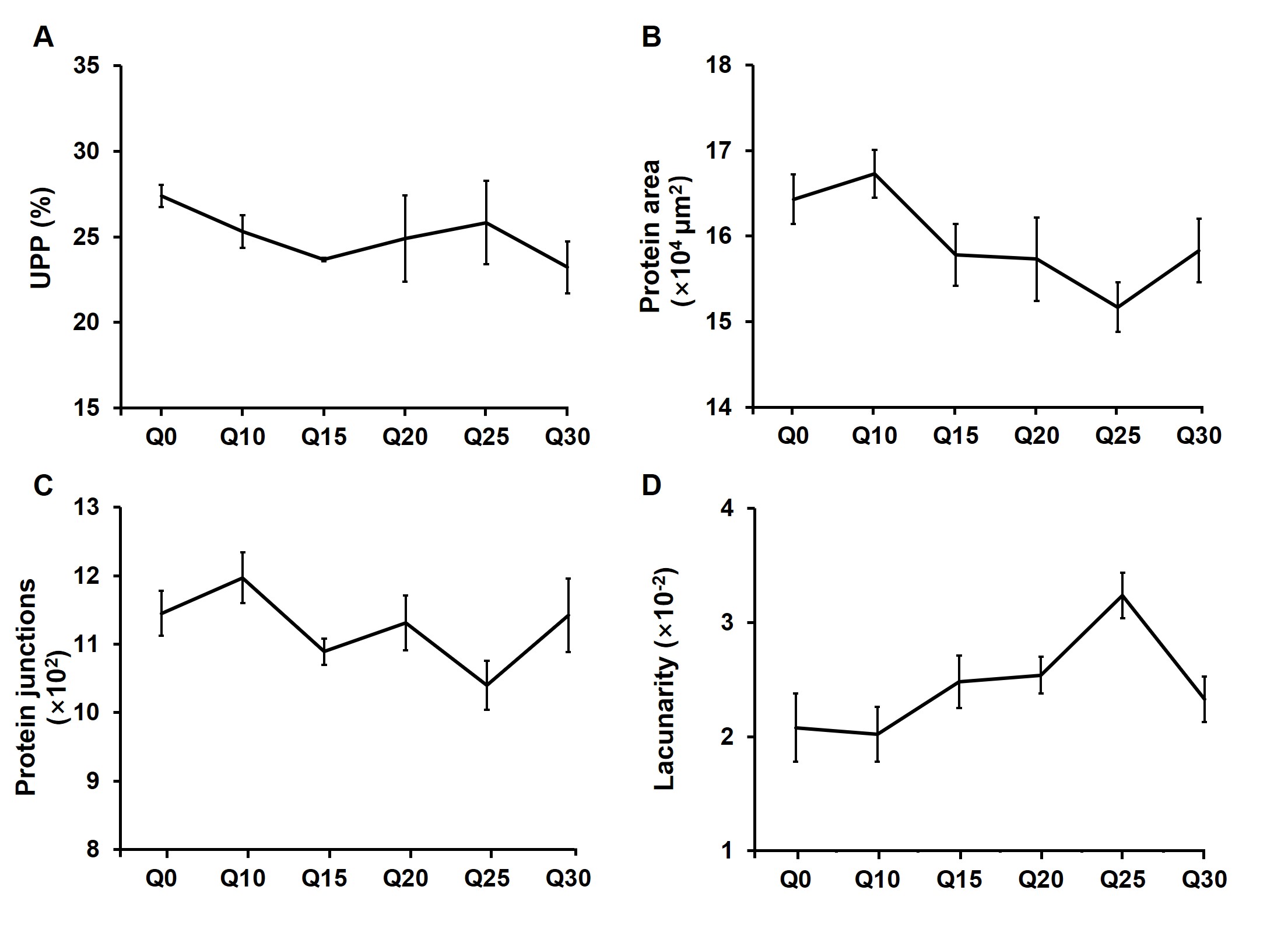

Supplement: Supplementary Figure 1 — Gluten protein composition and microstructure of wheat and quinoa reconstituted flours and doughs. (A) The UPP% of wheat and quinoa reconstituted flours. (B–D) Microstructure indicated by the protein area, protein junctions and lacunarity of the wheat and quinoa reconstituted doughs. The data were derived from five captures of each sample and presented as mean value ± standard deviation. [file Image_1.JPEG]

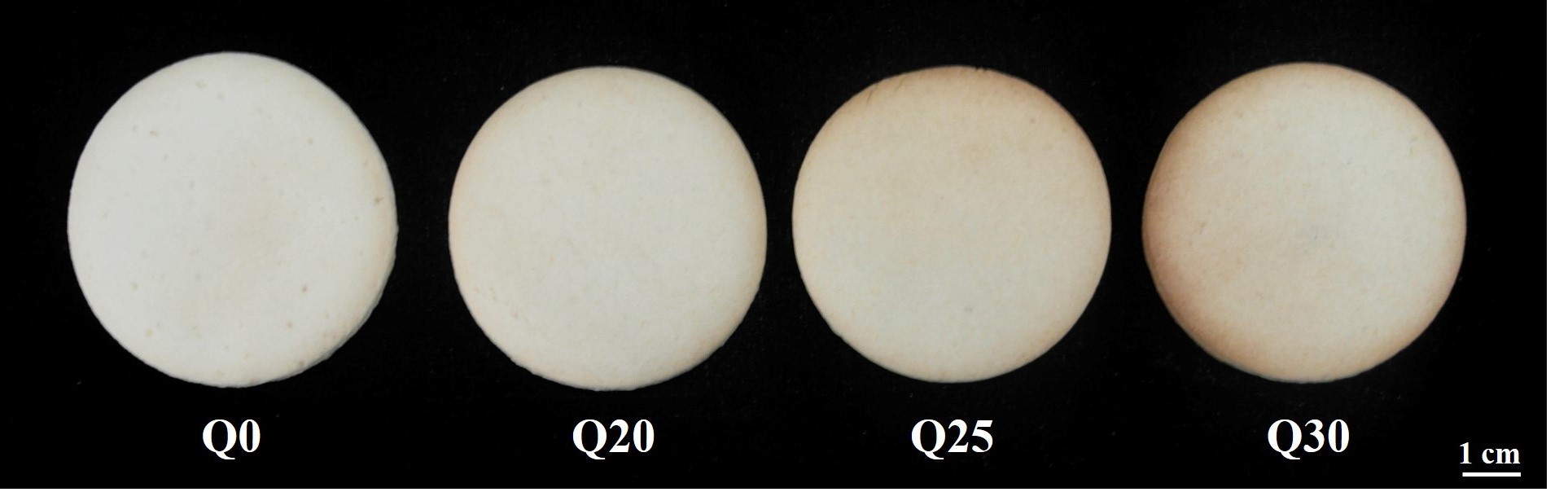

Supplement: Supplementary Figure 2 — Image of formulated crispy biscuits appearances and textures with the scale bar of 1 cm. [file Image_2.JPEG]

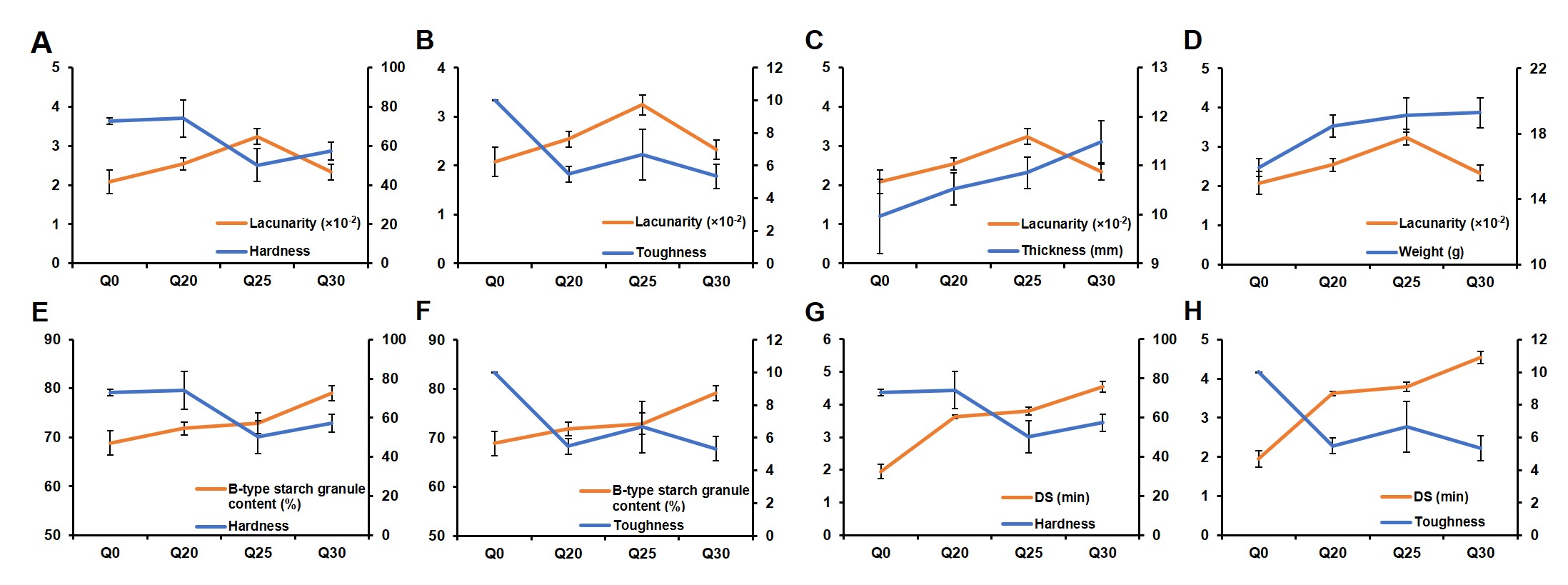

Supplement: Supplementary Figure 3 — The line charts exhibiting unobvious relationship between biscuit properties and gluten, starch and rheological properties of quinoa-wheat reconstituted systems. [file Image_3.JPEG]
